# Mesomorphic Behavior of Symmetric Azomethine Dimers Containing Different Chromophore Groups

**DOI:** 10.3390/molecules26082183

**Published:** 2021-04-10

**Authors:** Elena Perju, Luminita Marin

**Affiliations:** “Petru Poni” Institute of Macromolecular Chemistry of Romanian Academy, 700487 Iasi, Romania; lmarin@icmpp.ro

**Keywords:** symmetrical dimers, azomethines, thermotropic liquid crystals, mesomorphic glass, optical properties

## Abstract

A series of new azomethine dimers was synthesized by the condensation reaction of flexible bis-benzaldehydes with four aromatic amines containing phenyl, naphthyl, anthracene and pyrene groups. Their right structure was confirmed by FTIR and ^1^H-NMR spectroscopy and their thermal properties were investigated by thermogravimetric analysis, differential scanning calorimetry and polarized light optical microscopy. A view on their photophysical behavior was gained by UV-vis and photoluminescence spectroscopy. The dimers containing pyrene and anthracene presented liquid crystalline behavior, while the other dimers were crystalline compounds. Two dimers containing pyrene moieties formed mesomorphic glasses and had intense luminescence, promising properties for applications in building optoelectronic devices.

## 1. Introduction

Liquid crystal dimers are an important class of thermotropic compounds due to their specific behavior compared to conventional low molecular weight liquid crystals [1,2,3,4,5,6,7]. Initially considered as model compounds for semi-flexible main-chain polymers, they have developed over time as an independent class of compounds, between low molecular weight compounds and polymers, with subdivisions such as symmetric or non-symmetric dimers, and calamitic-, discotic- or bent-core-dimers, with particular thermotropic behavior [6,7,8,9,10,11,12,13]. Among these, symmetric liquid crystal dimers are formed from two identical mesogenic units linked by a flexible spacer. Their mesomorphic behavior is mainly influenced by the structure and length of mesogenic units, the nature, length and parity of flexible spacer, the terminal units and chains linked by mesogen cores, and also by the structure and position of linking groups between the mesogen units and the flexible spacer [14,15,16,17,18,19]. Due to their conjugation degree, liquid crystal dimers present not only opto-electronic properties but also the ability to self-organize in different continuous mesophases forming free defect films. Thus, they have proved useful for building optoelectronic devices such as wide-view displays, organic light emitting diodes, field effect transistors or solar cells [20,21]. In this context, the development of liquid crystal dimers with chromophoric mesogens is interesting for both fundamental science and practical applications [22,23].

In organic and macromolecular chemistry, the azomethine unit represents a versatile functional group due to its ability to support the self-organization towards different mesophases, but also to generate dynamic materials via covalent dynamic chemistry [24,25,26] while having high thermal stability, semiconducting properties and the ability to form organic complexes [27,28,29,30,31]. Due to this, azomethine linkage, also known as the imine or Schiff base, is frequently used in the building of new multifunctional liquid crystal dimers [12,32,33,34,35]. 

Polycyclic aromatic hydrocarbons such as naphthalene, anthracene and pyrene have been widely used in the development of functional materials with intense emission and unique charge transport properties. Incorporation of these chromophores in molecular materials facilitates π–π stacking and enhances charge migration improving the optical properties, due to the planar and rigid architecture of these segments [36,37,38,39,40,41]. Current interest in liquid crystalline dimers containing diether linked spacers with different lengths and parities, and π-conjugated mesogenic arm structures that tend to induce twist-bend nematic phases and nematic glassy states, was shown in [39,40,41,42,43].

In the present study we have attempted to combine the advantages associated with the azomethine linking group and chromophoric structures such as naphthalene, anthracene and pyrene in new symmetric liquid crystal dimers with emissive properties. For this aim, the chromophoric mesogenic units were connected via a poly(methylene)dioxy spacer. The liquid crystal properties of the new dimers were investigated using polarized light optical microscopy (PLM) and differential scanning calorimetry (DSC), while the emissive properties were evaluated by photoluminescence spectroscopy in solution. 

## 2. Results and Discussion 

### 2.1. Synthesis and Structural Characterization

**Scheme 1:**
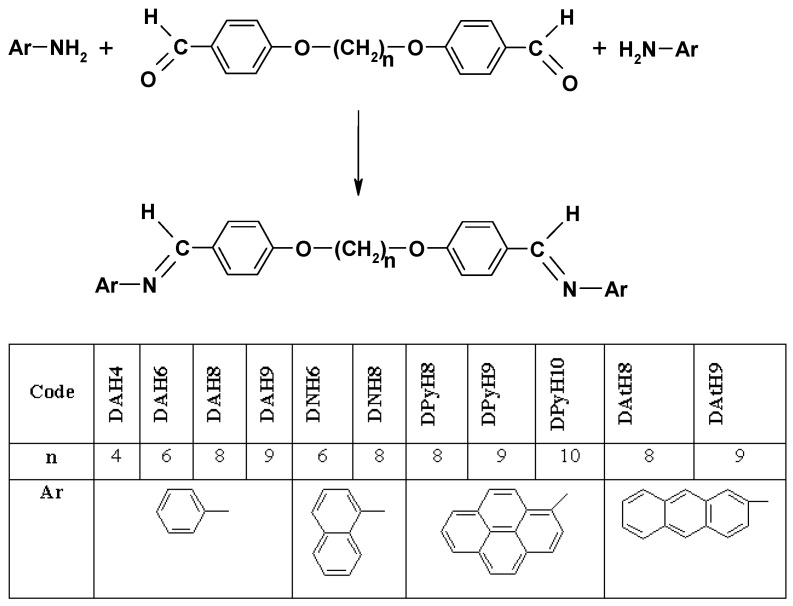
Synthesis of the azomethinic dimers (DArHn).

The symmetric azomethinic dimers (DArHn) were prepared via a condensation reaction of 4,4’-diformyl-diphenoxyalcanes (Hn) with different aromatic amines (Ar-NH2) (aniline, 1-naphthylamine, 2-aminoanthracene and 1-aminopyrene) in a ½ molar ratio resulting in eleven symmetric dimers, as shown in Scheme 1. 

**Scheme 2:**
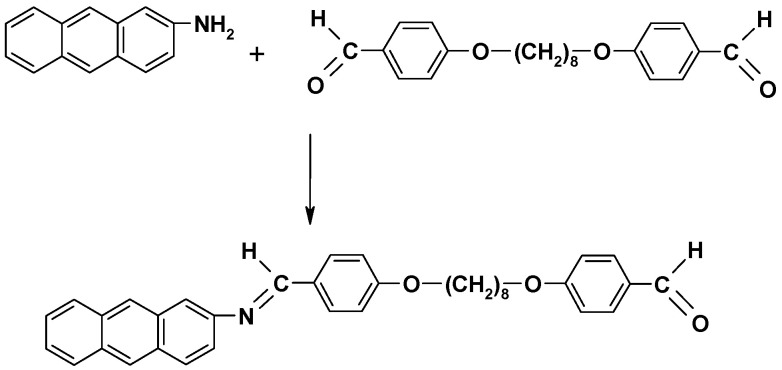
Synthesis of the azomethinic model compound (MAtH8).

As the anthracene dimers showed poor solubility, which hampered their structure characterization, a mono-azomethine (MAtH8) was synthetized as a model compound, by reacting 4,4’-diformyl-diphenoxyoctane with 2-aminoanthracene in a 1/1 molar ratio, in similar conditions (Scheme 2).

The expected structures of the DArHn dimers were confirmed by FTIR spectroscopy through the appearance of a characteristic absorption band, at 1618–1630 cm^−1^, due to the new azomethine linkages formed by the condensation reaction, while the absorption bands distinctive for aldehyde and amine groups disappeared. Moreover, the FTIR spectra displayed specific absorption bands for the stretching vibrations of ether linkage, aromatic units and aliphatic chains, while the MAtH8 mono-azomethine compound additionally showed an absorption band at 1683 cm^−1^ corresponding to the unreacted carbonyl group. Detailed FTIR characterization is provided in the experimental part.

**Figure 1:**
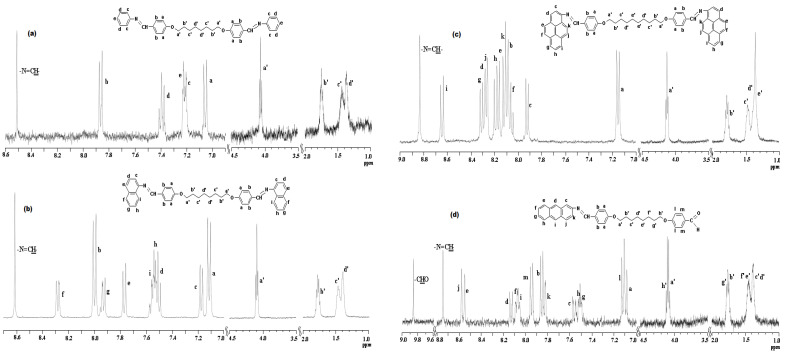
Representative ^1^H-NMR spectra of (**a**) DAH8, (**b**) DNH8, (**c**) DPyH10 and (**d**) MAtH8.

The ^1^H-NMR spectra confirmed the FTIR results by the appearance of characteristic chemical shifts for the azomethine, aromatic and aliphatic protons, in the right ratio of their integrals (Figure 1a–d). The ^1^H-NMR spectra of the anthracene-containing dimers could not be recorded due to their poor solubility in deuterated solvents. However, the ^1^H-NMR spectra of the MAtH8 model compound displayed chemical shifts characteristic to the targeted mono-azomethine, confirming the formation of an azomethine linkage between anthracene and aromatic aldehyde. Moreover, the FTIR spectra of the model compound, except the carbonyl band, was similar to that of the corresponding dimers, confirming once more the right structure of the anthracene-based dimers.

The solubility of the azomethine dimers (DArHn) and model compound (MAtH8) was tested in different organic solvents such as *N*, *N*-dimethylformamide (DMF), dimethylsulfoxide (DMSO), tetrahydrofuran (THF) and chloroform (CHCl_3_). The dimers containing phenyl and naphthyl moieties were completely soluble at heating in all the solvents, while the dimers containing pyrene moieties were soluble in THF and chloroform at room temperature, and in DMF and DMSO at heating. The dimers containing anthracene moieties had poor solubility, being soluble in high polar solvents at heating, while in less polar solvents they were insoluble (Table 1).

### 2.2. Thermal Properties

#### 2.2.1. Thermal Stability

**Figure 2:**
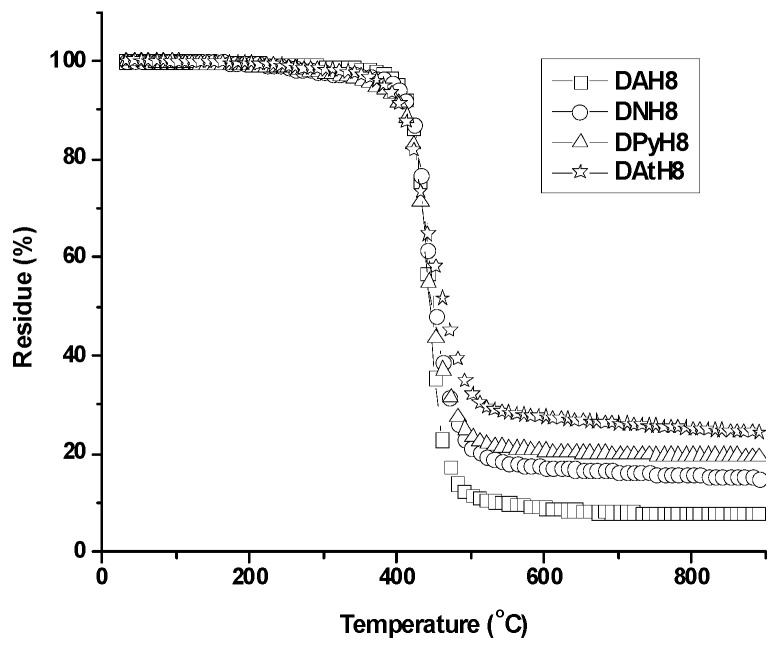
Representative TGA traces of the azomethine dimers.

The thermal stability of the dimers was investigated by thermogravimetric analysis (TGA), at a heating rate of 10 °C/min, in an inert atmosphere. As a first observation, all the dimers displayed quite similar thermogravimetric curves (Figure 2), with the decomposition onset at 330–376 °C, and 10% weight loss at temperatures above 415 °C. They showed a maximum rate of weight loss at temperatures from 433 to 445 °C, reaching a low maximum residue yield at 900 °C, i.e., 8% for the dimer containing phenyl units and eight methylene units in the flexible spacer, and 15–24% for the other dimers. These thermal data indicate that the dimers are highly thermostable, allowing practical processing and possible use in devices [44].

#### 2.2.2. Thermotropic Behavior

The thermotropic behavior of azomethine dimers (DArHn) and model compound (MAtH8) was monitored by differential scanning calorimetry (DSC) and polarized light optical microscopy (PLM). The transition temperatures recorded by both methods, with a heating/cooling rate of 10 °C/min, are presented in Table 2. 

The dimers containing phenyl (DAHn) and naphthyl (DNHn) moieties did not show any mesophase in PLM. The DAHn dimers showed only a melting process during the heating and a crystallization process during the cooling. This behavior was confirmed by DSC curves, which showed one endothermic peak in the DSC heating scan, corresponding to the melting temperature and an exothermic peak in the DSC cooling scan, assigned to the crystallization temperature. The enthalpy of both endothermic and exothermic events had high values, characteristic for these with a large difference in their ordering degree (tridimensional ordered crystalline state and isotropic state). The DNHn dimers presented two endothermic peaks in the DSC heating scan assigned by PLM to polymorphic transitions, and a glass transition in the cooling scan. Even if the crystallization was not observed in the DSC traces, PLM revealed a slow crystallization process occurring after several days. Thus, it was concluded that the dimers containing phenyl and naphthyl moieties do not organize in mesophases during heating/cooling, and consequently they are not liquid crystals.

**Figure 3:**
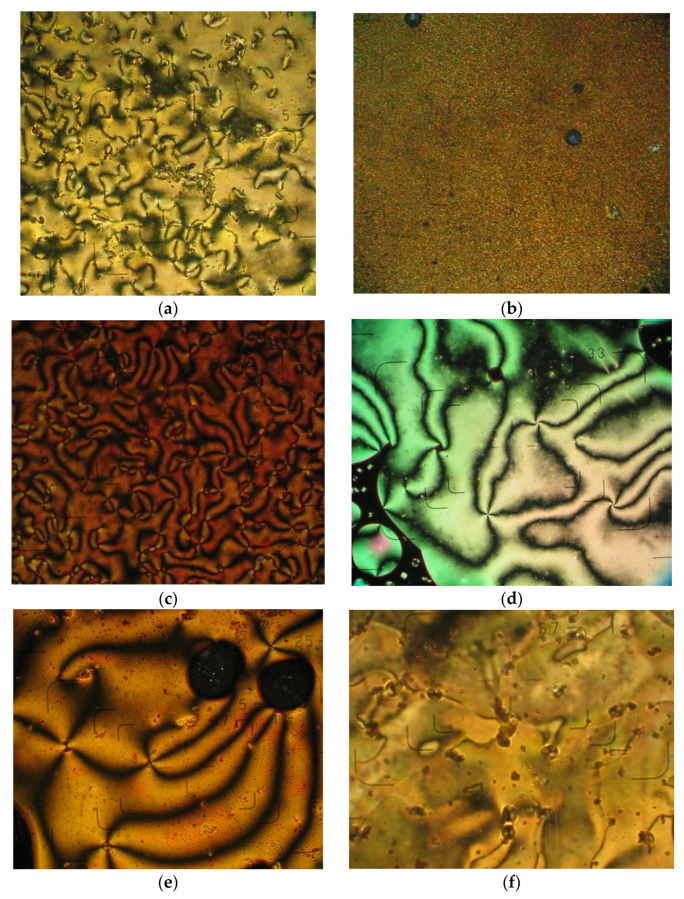
Microphotographs of the azomethine dimers DPyH8 (2C, 157 °C) (**a**), DPyH9 (1C, 106 °C) (**b**), DPyH10 (1C, 132 °C) (**c**), MAtH8 (2C, 242 °C) (**d**), DAtH8 (1C, 244 °C) (**e**) and DAtH9 (1C, 250 °C) (**f**), 1C—first cooling scan, 2C—second cooling scan, in optical light microscopy, crossed polarizers.

The dimers containing pyrene (DPyHn) and anthracene (DAtHn) units showed monotropic nematic liquid crystalline behavior, except the DAtH9 dimer and the MAtH8 model compound, which presented an enantiotropic behavior. Thus, DPyH10, MAtH8 and DAtH8 revealed crystalline melting during the heating cycles, while during the cooling cycles they showed the occurrence of a Schlieren nematic texture from the isotropic fluid, which passed in a marble texture in case of DPyH8 (Figure 3a,c,d,f). A similar behavior was observed for DPyH9, with the difference that the texture appearing from the isotropic phase was a fine granular one (Figure 3b) [45]. DAtH9 showed the transformation of the crystalline state into a marble texture during heating, while during the cooling the appearance of nematic droplets formed by coalescence was noted, a Schlieren type texture that further passed in a marble one (Figure 3f). The nematic texture of the dimers containing pyrene moieties froze, forming a mesomorphic glass without cracks, which was stable over the three months of investigation [35,46]. Those containing anthracene units showed the transformation of the nematic texture into the crystalline state. 

**Figure 4:**
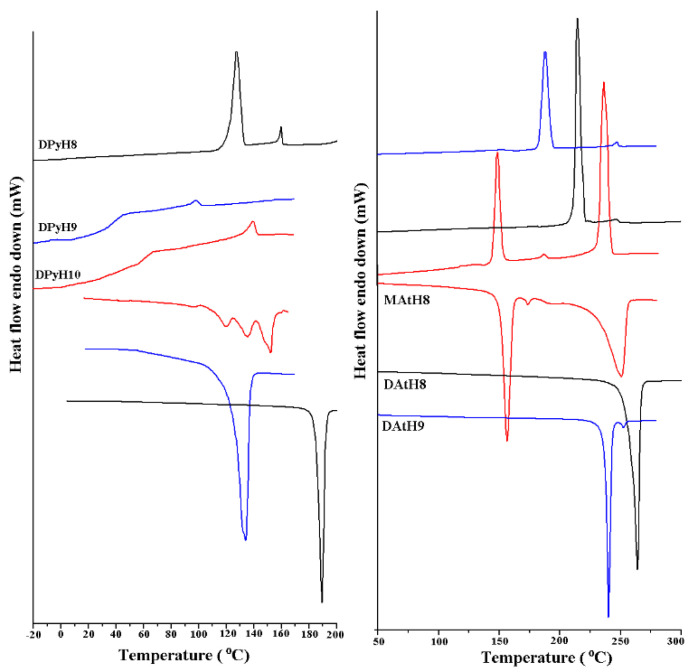
Differential scanning calorimetry (DSC) traces of the azomethine dimers (DPyHn and DAtHn) and model compound (MAtH8).

The liquid crystal behavior observed by polarized light optical microscopy was confirmed by differential scanning calorimetry. In the first heating scan, the DSC curves of the monotropic liquid crystalline dimers showed one endotherm corresponding to the crystalline–isotropic transition, except for the DPyH10 and MAtH8, which showed multiple endotherms attributed by PLM to crystalline–crystalline transitions. In the second heating scan, in the same region, there appeared only a single endotherm and a glass transition. In the cooling scan, the monotropic liquid crystalline dimers presented one sharp exothermic peak corresponding to the isotropic–nematic (I–N) transition (Figure 4). The dimers containing anthracene units also showed a second exothermic peak corresponding to the nematic–crystalline (N–I) transition, while no such an exotherm was recorded for the pyrene-containing dimers, in accordance with the PLM observation of the texture freezing. The DSC curves of the enantiotropic DAtH9 liquid crystal showed two endothermic peaks in the heating scans corresponding to crystalline–nematic and nematic–isotropic transitions, and two exothermic peaks in the cooling scan corresponding to the isotropic–nematic and nematic–crystalline transitions. In the case of the azomethinic compound (MAtH8), in both the heating and cooling scans three maximum peaks were recorded, of which the first peak corresponded to a crystalline–crystalline transition, the second peak was assigned to a liquid crystalline–nematic transition and the third peak correlated to a nematic–isotropic transition. As expected, the enthalpy of the nematic–isotropic and isotropic–nematic transitions had lower values compared to that of the crystalline–nematic and nematic–crystalline ones, in line with the lower energy involved in the transition between two states with closer ordering degrees [47]. The exception to this rule was the MAtH8 model compound, which showed quite high transition enthalpy, possibly due to an easier alignment of the molecules into a nematic mesophase favored by a greater mobility, thus developing stronger intermolecular forces. Looking at the data in Table 2, it can be remarked that the thermotropic behavior of the dimers was influenced by the flexible spacer length and mesogen length as well. Thus, the mesophase appearance, melting and isotropic temperature increased along with the increasing of the mesogen length and decreased with increasing the flexible spacer length, while the stability range of the nematic mesophase increased. An odd–even effect, consisting of lower transition temperatures for the dimers containing odd flexible spacers, was noted [48,49].

### 2.3. Optical Properties

The studied compounds contain chromophore units that should impact the ordering ability and photophysical properties as well. In this view, the optical properties of the azomethine dimers (DArHn) and model compound (MAtH8) were investigated by UV-Vis and photoluminescence (PL) spectroscopy on 10^−4^ M dimethylformamide solutions. The results are summarized in Table 3.

**Figure 5:**
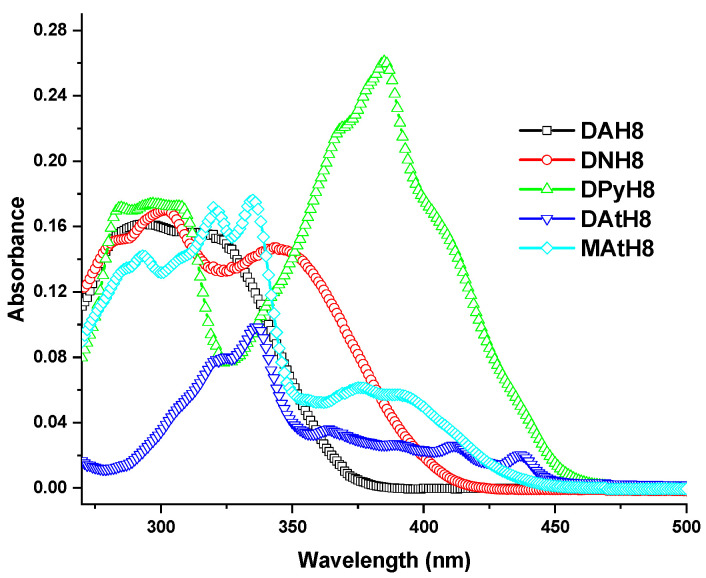
UV-Vis spectra of the azomethine dimers (DArH8) and model compound (MAtH8) containing eight methylene groups in the flexible spacer, in DMF solutions.

The absorption spectra of the azomethine dimers in solution are displayed in Figure 5. All the dimers showed a complex absorption profile, with two main broad absorption bands composed from numerous shoulders, attributable to the high number of different conformations of the cromophoric units raised by the intramolecular motions in solution [50]. It is expected that the first absorption band is promoted by the π-π* electronic transitions of the phenylene ring, including the phenylene-imine frame [51,52,53], while the second broad absorption band can be assigned to the polycyclic aromatic groups (naphtyl, anthracene or pyrene) and the frame resulting from their conjugation with the imine linkage. As expected, a bathochromic shift of the absorption bands can be noted along with the increase of the number of fused aromatic rings [54].

It can clearly be seen that the number of fused aromatic rings influenced the values of the energy gap (Table 3). It is evident from these values that the energy gap between the highest energy bonding π-orbital (HOMO) and the lowest energy anti-bonding π^∗^-orbital (LUMO) decreased as the number of aromatic fused rings increased, reaching values of 2.63 eV for the pyrene-containing dimers and 2.66 eV for the anthracene-based dimers. These band-gap values are typical for organic semiconductors and indicate that less energy is required to carry out the electron promotion [55]. However, the pyrene-containing dimers showed higher molar absorptivity coefficients indicating a higher probability of the electronic transitions, correlating with the more planar π-electron delocalized structure [56]. Interestingly enough, an odd–even effect of the absorption coefficient of the dimers containing pyrene or anthracene chromophores was observed, consisting of a significantly higher absorption coefficient for the odd members compared to the even ones. This can possibly be correlated with the twisting promoted by the odd number of carbons in the flexible spacer [57], but we have no clear explanation at this moment. All in all, these two parameters indicate the pyrene-containing dimers as the most suitable for opto-electronic applications.

**Figure 6:**
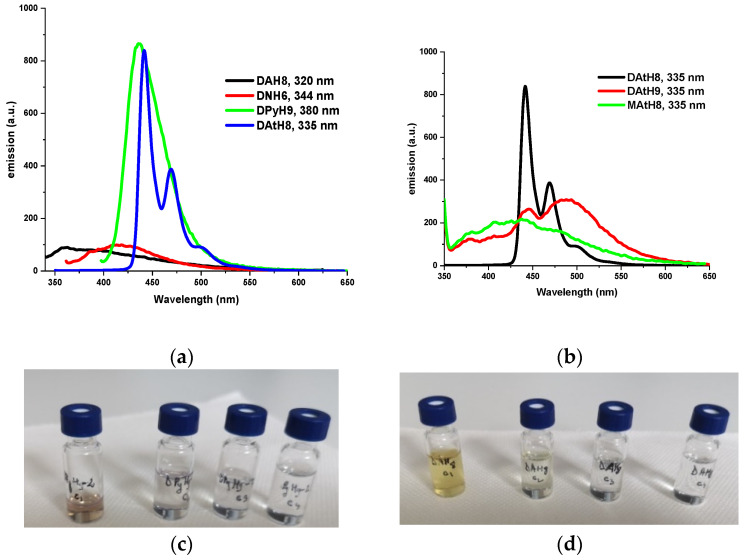

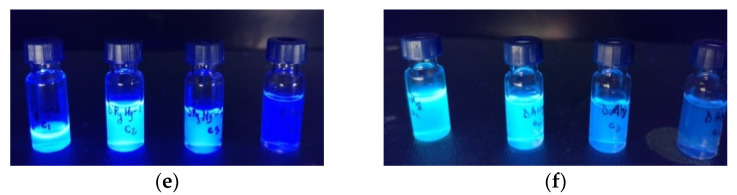
Photoluminescence spectra of the azomethine dimers, in DMF solutions, when excited with the absorption maximum (given in inset) (**a**,**b**) and photographs of the DPyH9 and DAtH8 solutions under daylight (**c**,**d**) and UV light (365 nm) (**e**,**f**).

The photoluminescence properties of the studied dimers were investigated by excitation at the maximum absorption wavelengths characteristic of the chromophore-imine frame. The dimers containing phenyl or naphthalene moieties presented a broad emission band in the UV and violet domains, respectively, while the dimers containing pyrene units showed a sharp emission band in the blue domain (Figure 6a). The photoluminescence spectra of the anthracene-containing dimers showed superposed emission bands, indicative of less electron delocalization leading to more fluorophore units [56,58]. Thus, the DAtH9 dimer and the MAtH8 model compound presented a broad emission band with four peaks in UV, violet, blue and green domains, while the DAtH8 dimer showed a sharp emission band with three maxima: The first two in the blue domain and the last one in the green domain (Figure 6b).

Interestingly enough, by illumination with a UV lamp, the DMF solutions of dimers containing anthracene and pyrene fluorphores showed a blue light emission, whose intensity increased as the concentration increased (Figure 6e,f). This behavior, opposite to the well-known quenching emission effect, indicates a possible aggregation-induced emission enhancement (AIEE), a promising photophysical behavior for real-life applications [59]. Considering the ability of the dimers to form liquid crystal mesophases, which allow the manufacture of ordered continuous films and their ability to emit light, it is envisaged that these compounds are of further interest for the preparation of active substrates for optoelectronic devices [60,61]. 

## 3. Materials and Methods

### 3.1. Characterization

Infrared spectra were recorded on a FTIR Bruker Vertex 70 Spectrophotometer (Ettlingen, Germany) in the transmission mode, by using KBr pellets. Proton nuclear magnetic resonance spectra were recorded with a Bruker Advance DRX 400 MHz spectrometer (Rheinstetten, Germany) using DMSO-d6 as the solvent, and chemical shifts are reported in parts per million (ppm).

Thermogravimetric analysis was carried out using a Jupiter STA 449F1 derivatograph (Selb, Germany), at a heating rate of 10 °C/min, under a nitrogen atmosphere, from room temperature to 900 °C. The temperature corresponding to the onset on the TG curve was regarded as the initial decomposition temperature (T_0_). The maximum decomposition rate temperature, which is the maximum signal in the differential thermogravimetry (DTG) curve, was also recorded. 

DSC measurements were performed with a Pyris Diamond DSC, Perkin Elmer (Shelton, CT, USA) system, under a nitrogen atmosphere. PLM observations were carried out with an Olympus BH-2 polarized light microscope (Center Valley, PA, USA) under cross-polarizers with a temperature control system THMS 600/HSF9I hot stage. For all measurements a 20x objective lens was used. The eyepiece had 10x magnification.

UV-Vis absorption and photoluminescence spectra were recorded on a Carl Zeiss Jena SPECORD M42 spectrophotometer (Jena, Germany) and a Perkin Elmer LS 55 spectrophotometer (Beaconsfield, UK), respectively, in very diluted solutions, using 10 mm quartz cells fitted with poly(tetrafluoroethylene) stoppers.

Solubility tests of the dimers were performed in common solvents such as *N*,*N*-dimethylformamide, dimethylsulfoxide, tetrahydrofuran and chloroform.

### 3.2. Reagents

Aniline, 1-naphthylamine, 2-aminoanthracene, 1-aminopyrene and *N*,*N*-dimethylformamide were purchased from Sigma-Aldrich Chemie GmbH (Germany) and used as received. α,ω-bis(4-formylphenoxy)-alcanes: (1,4-bis(4-formylphenoxy)-butane; 1,6-bis(4-formyl phenoxy)-sextane, 1,7-bis(4-formylphenoxy)-heptane, 1,8-bis(4-formylphenoxy)-octane, 1,9-bis(4-formylphenoxy)-nonane, 1,10-bis(4-formylphenoxy)-decane) were prepared and structurally characterized in our laboratory according to published procedures [51,62].

### 3.3. Synthetic Procedure

The synthesis of the azomethine dimers (DArHn) was performed by a condensation reaction of 4,4’-diformyl-diphenoxyalcanes (Hn) with different aromatic amines (Ar-NH_2_) (aniline, 1-naphthylamine, 2-aminoanthracene and 1-aminopyrene) in a 1:2 molar ratio. In order to avoid mono-reacted compound formation, the amines were used in 10% excess. The azomethine model compound (MAtH8) was synthesized by a condensation reaction of 1,8-bis(4-formylphenoxy)-octane with 2-aminoanthracene in an equimolecular ratio. The reagents were dissolved in DMF to form a 15% solution and the reaction mixtures were stirred for 16 to 90 h, at 110 °C, under a nitrogen atmosphere. After 8 h of stirring, a few drops of acetic acid were added as a catalyst. After cooling, the precipitated products were filtered, washed with methanol or *N*,*N*-dimethylformamide, then hot filtered, and the soluble dimers were recrystallized from DMF. Finally, the compounds were dried in a vacuum for 24 h at 60 °C.

*1,4-Bis[4-(phenyliminomethylidene)-phenoxy]-butane*

FTIR (KBr, ν, cm^−1^): 3049 (=C–H stretch of the aromatic rings), 2950–2846 (C–H stretch of aliphatic chains), 1623 (–N=CH– stretch), 1607, 1574, 1514 (C–C ring stretch), 1250, 1166 (C–O–C– stretch), 820 (absorption band assigned to 1,4-phenylene ring). ^1^H-NMR (d-DMSO, δ, ppm): 8.52 (s, 2H, N=CH), 7.09; 7.07 (d, 4H, ortho to –O–), 7.89, 7.87 (d, 4H, ortho to –CH=N–), 7.23, 7.21 (d, 4H, ortho to –N=CH–), 7.23–7.20 (t, 2H, para to –N=CH–), 7.41–7.38 (t, 4H, metha to –N=CH–), 4.17–4.14 (t, 4H, α to Ar–O–), 1.93 (m, 4H, β to Ar–O–).

*1,6-Bis[4-(phenyliminomethylidene)-phenoxy]-hexane*

FTIR (KBr, ν, cm^−1^): 3059 (=C–H stretch of the aromatic rings), 2940–2866 (C–H stretch of aliphatic chains), 1620 (–N=CH– stretch), 1606, 1573, 1507 (C–C ring stretch), 1244, 1165 (C–O–C– stretch), 836 (absorption band assigned to 1,4-phenylene ring). ^1^H-NMR (d-DMSO, δ, ppm): 8.51 (s, 2H, N=CH), 7.07, 7.00 (d, 4H, ortho to –O–), 7.88, 7.86 (d, 4H, ortho to –CH=N–), 7.23, 7.21 (d, 4H, ortho to –N=CH–), 7.22–7.19 (t, 2H, para to –N=CH–), 7.41–7.38 (t, 4H, metha to –N=CH–), 4.09–4.06 (t, 4H, α to Ar–O–), 1.78 (m, 4H, β to Ar–O–), 1.51 (m, 4H, γ to Ar–O–).

*1,8-Bis[4-(phenyliminomethylidene)-phenoxy]-octane*

FTIR (KBr, ν, cm^−1^): 3059 (=C–H stretch of the aromatic rings), 2937–2857 (C–H stretch of aliphatic chains), 1620 (–N=CH– stretch), 1606, 1573, 1507 (C–C ring stretch), 1245, 1166 (C–O–C– stretch), 836 (absorption band assigned to 1,4-phenylene ring). ^1^H-NMR (d-DMSO, δ, ppm): 8.51 (s, 2H, N=CH), 7.06, 7.04 (d, 4H, ortho to –O–), 7.87, 7.85 (d, 4H, ortho to –CH=N–), 7.23, 7.21 (d, 4H, ortho to –N=CH–), 7.23–7.20 (t, 2H, para to –N=CH–), 7.41–7.37 (t, 4H, metha to –N=CH–), 4.07–4.04 (t, 4H, α to Ar–O–), 1.78–1.73 (m, 4H, β to Ar–O–), 1.46 – 1.38 (m, 8H, γ and δ to Ar–O–).

*1,9-Bis[4-(phenyliminomethylidene)-phenoxy]-nonane*

FTIR (KBr, ν, cm^−1^): 3054 (=C–H stretch of the aromatic rings), 2932–2851 (C–H stretch of aliphatic chains), 1622 (–N=CH– stretch), 1605, 1571, 1510 (C–C ring stretch), 1247, 1165 (C–O–C– stretch), 836 (absorption band assigned to 1,4-phenylene ring). ^1^H-NMR (d-DMSO, δ, ppm): 8.51 (s, 2H, N=CH), 7.06, 7.04 (d, 4H, ortho to –O–), 7.87, 7.85 (d, 4H, ortho to –CH=N–), 7.23, 7.21 (d, 4H, ortho to –N=CH–), 7.23–7.20 (t, 2H, para to –N=CH–), 7.41–7.37 (t, 4H, metha to –N=CH–), 4.06–4.03 (t, 4H, α to Ar–O–); 1.77 – 1.70 (m, 4H, β to Ar–O–), 1.43 – 1.34 (m, 10H, γ, δ and ε to Ar–O–).

*1,6-Bis[4-(naphthyliminomethylidene)-phenoxy]-hexane*

FTIR (KBr, ν, cm^−1^): 3047 (=C–H stretch of the aromatic rings), 2941–2856 (C–H stretch of aliphatic chains), 1618 (–N=CH– stretch), 1605, 1565, 1513 (C–C ring stretch), 1246, 1166 (C–O–C– stretch), 772 (absorption band assigned to 1,4-phenylene ring). ^1^H-NMR (d-DMSO, δ, ppm): 8.62 (s, 2H, N=CH), 7.13, 7.11 (d, 4H_a_), 8.01, 7.99 (d, 4H_b_), 7.19, 7.17 (d, 2H_c_), 7.51–7.49 (t, 2H_d_), 7.56–7.53 (t, 2H_h_), 7.57–7.54 (t, 2H_i_), 7.78, 7.76 (d, 2H_e_), 7.94, 7.92 (d, 2H_g_), 8.29, 8.27 (d, 2H_f_), 4.12–4.09 (t, 4H, α to Ar–O–), 1,81 (m, 4H, β to Ar–O–), 1.54 (m, 4H, γ to Ar–O–)

*1,8-Bis[4-(naphthyliminomethylidene)-phenoxy]-octane*

FTIR (KBr, ν, cm^−1^): 3046 (=C–H stretch of the aromatic rings), 2941–2851 (C–H stretch of aliphatic chains), 1628 (–N=CH– stretch), 1599, 1572, 1511 (C–C ring stretch), 1252, 1164 (C–O–C– stretch), 772 (absorption band assigned to 1,4-phenylene ring). ^1^H-NMR (*d*-DMSO, δ, ppm): 8.61 (s, 2H, N=CH), 7.12, 7.10 (*d*, 4H_a_), 8.00, 7.98 (*d*, 4H_b_), 7.19, 7.17 (*d*, 2H_c_), 7.51–7.49 (t, 2H_d_), 7.56–7.53 (t, 2H_h_), 7.57–7.54 (t, 2H_i_), 7.77, 7.75 (*d*, 2H_e_), 7.95, 7.93 (*d*, 2H_g_), 8.29, 8.27 (*d*, 2H_f_), 4.10–4.07 (t, 4H, α to Ar–O–), 1.81–1.74 (m, 4H, β to Ar–O–), 1.47–1.39 (m, 8H, γ and δ to Ar–O–).

*1,8-Bis[4-(pyreneiminomethylidene)-phenoxy]-octane*

FTIR (KBr, ν, cm^−1^): 3040 (=C–H stretch of the aromatic rings), 2932–2853 (C–H stretch of aliphatic chains), 1628 (–N=CH– stretch), 1603, 1588, 1571 (C–C ring stretch), 1252, 1163 (C–O–C– stretch), 841 (absorption band assigned to 1,4-phenylene ring). ^1^H-NMR (*d*-DMSO, δ, ppm): 8.84 (s, 2H, N=CH), 7.17, 7.15 (*d*, 4H_a_), 8.11, 8.09 (*d*, 4H_b_), 7.93, 7.91 (*d*, 2H_c_), 8.08, 8.06 (*d*, 2H_f_), 8.12, 8.10 (*d*, 2H_k_), 8.17, 8.15 (*d*, 2H_e_), 8.20, 8.18 (*d*, 2H_h_), 8.27, 8.25 (*d*, 2H_j_), 8.29, 8.27 (*d*, 2H_d_), 8.31, 8.29 (*d*, 2H_g_), 8,65, 8.63 (*d*, 2H_i_), 4.14–4.11 (t, 4H, α to Ar–O–), 1.83–1.78 (m, 4H, β to Ar–O–), 1.50–1.42 (m, 8H, γ and δ to Ar–O–).

*1,9-Bis[4-(pyreneiminomethylidene)-phenoxy]-nonane*

FTIR (KBr, ν, cm^−1^): 3038 (=C–H stretch of the aromatic rings), 2933–2851 (C–H stretch of aliphatic chains), 1627 (–N=CH– stretch), 1601, 1587, 1511 (C–C ring stretch), 1243, 1163 (C–O–C– stretch), 843 (absorption band assigned to 1,4-phenylene ring). ^1^H-NMR (*d*-DMSO, δ, ppm): 8.82 (s, 2H, N=CH), 7.16, 7.14 (*d*, 4H_a_), 8.10, 8.08 (*d*, 4H_b_), 7.92, 7.90 (*d*, 2H_c_), 8.05, 8.03 (*d*, 2H_f_), 8.12, 8.10 (*d*, 2H_k_), 8.17, 8.15 (*d*, 2H_e_), 8.19, 8.17 (*d*, 2H_h_), 8.27, 8.25 (*d*, 2H_j_), 8.29, 8.27 (*d*, 2H_d_), 8.31, 8.29 (*d*, 2H_g_), 8.65, 8.63 (*d*, 2H_i_), 4.12–4.09 (t, 4H, α to Ar–O–), 1.80–1.75 (m, 4H, β to Ar–O–), 1.48–1.38 (m, 10H, γ, δ and ε to Ar–O–)

*1,10-Bis[4-(pyreneiminomethylidene)-phenoxy]-decane*

FTIR (KBr, ν, cm^−1^): 3037 (=C–H stretch of the aromatic rings), 2922, 2850 (C–H stretch of aliphatic chains), 1627 (–N=CH– stretch), 1601, 1590, 1509 (C–C ring stretch), 1251, 1164 (C–O–C– stretch), 839 (absorption band assigned to 1,4-phenylene ring). ^1^H-NMR (*d*-DMSO, δ, ppm): 8.83 (s, 2H, N=CH), 7.16, 7.14 (*d*, 4H_a_), 8.10, 8.08 (*d*, 4H_b_), 7.92, 7.93 (*d*, 2H_c_), 8.05, 8.03 (*d*, 2H_f_), 8.12, 8.10 (*d*, 2H_k_), 8.17, 8.15 (*d*, 2H_e_), 8.19, 8.17 (*d*, 2H_h_), 8.27, 8.25 (*d*, 2H_j_), 8.29, 8.27 (*d*, 2H_d_), 8.31, 8.29 (*d*, 2H_g_), 8.65, 8.63 (*d*, 2H_i_), 4.12–4.09 (t, 4H, α to Ar–O–), 1.80–1.75 (m, 4H, β to Ar–O–), 1.47–1.35 (m, 12H, γ, δ and ε to Ar–O–).

*1,8-Bis[4-(anthraceneiminomethylidene)-phenoxy]-octane*

FTIR (KBr, ν, cm^−1^): 3049 (=C–H stretch of the aromatic rings), 2936–2856 (C–H stretch of aliphatic chains), 1630 (–N=CH– stretch), 1604, 1576, 1510 (C–C ring stretch), 1248, 1163 (C–O–C– stretch), 896 (absorption band assigned to 1,4-phenylene ring).

*1,9-Bis[4-( anthraceneiminomethylidene)-phenoxy]-nonane*

FTIR (KBr, ν, cm^−1^): 3049 (=C–H stretch of the aromatic rings), 2923, 2849 (C–H stretch of aliphatic chains), 1624 (–N=CH– stretch), 1598, 1571, 1509 (C–C ring stretch), 1249, 1162 (C–O–C– stretch), 897 (absorption band assigned to 1,4-phenylene ring).

*α-[4-(2- anthraceneiminomethylidene)-phenoxy]-ω-[4-oxybenzaldehyde]-octane*

FTIR (KBr, ν, cm^−1^): 3045 (=C–H stretch of the aromatic rings), 2936 – 2856 (C–H stretch of aliphatic chains), 1683 (–CH=O), 1630 (–N=CH– stretch), 1604, 1576, 1509 (C–C ring stretch), 1248, 1163 (C–O–C– stretch), 895 (absorption band assigned to 1,4-phenylene ring). ^1^H-NMR (*d*-DMSO, δ, ppm): 9.86 (s, 1H, CH=O), 8.74 (s, 2H, N=CH), 7.11, 7.09 (*d*, 2H_a_), 7.13, 7.11 (*d*, 2H_l_), 7.86, 7.84 (*d*, 2H_b_), 7.95, 7.93 (*d*, 2H_m_), 7.52, 7.50 (*d*, 1H_g_), 7.53, 7.51 (*d*, 1H_h_), 7.57, 7.55 (*d*, 1H_c_), 7.82 (s, 1H_k_), 8.07, 8.05 (*d*, 1H_i_), 8.09, 8.07 (*d*, 1H_f_), 8.14, 8.12 (*d*, 1H_d_), 8.55 (s, 1H_e_), 8.58 (s, 1H_j_), 4.09–4.06 (t, 2H_a’_); 4.11–4.07 (t, 2H_h’_), 1.79–1.74 (m, 4H_b’+ f’_), 1.45–1.38 (m, 8H _c’+ g’+ d’+ h’_).

## 4. Conclusions

Four series of azomethine dimers containing different chromophore groups (phenyl, naphthyl, pyrene and anthracene) linked by a flexible spacer were synthesized by acid condensation. The obtained azomethine dimers had high thermal stability and relatively good solubility in polar solvents. The dimers containing anthracene and pyrene chromophores displayed thermotropic behavior, with nematic monotropic or enantiotropic mesophases. The melting and isotropic temperatures increased with the length of mesogenic core and decreased with the number of methylene groups of the flexible spacer. Two dimers containing pyrene moieties vitrified in a mesomorphic state forming mesomorphic glasses, which are useful for the preparation of ordered continuous films as active substrates in optoelectronic applications. Furthermore, the presence of pyrene led to a high absorption coefficient and intense luminescence, promising good potential for optoelectronic applications.

## Figures and Tables

**Table 1:** Solubility characteristics of the azomethinic dimers (DArHn) and compound (MAtH8).

Code	DAH4	DAH6	DAH8	DAH9	DNH6	DNH8	DPyH8	DPyH9	DPyH10	DAtH8	DAtH9	MAtH8
**DMSO**	(+)	(+)	(+)	(+)	(+)	(+)	(+)	(+)	(+)	(+)	(+)	(+)
**DMF**	(+)	(+)	(+)	(+)	(+)	(+)	(+)	(+)	(+)	(+)	(+)	(+)
**CHCl**_**3**_	(+)	(+)	(+)	(+)	(+)	(+)	+	+	+	(−)	(−)	(−)
**THF**	(+)	(+)	(+)	(+)	(+)	(+)	+	+	+	(±)	(±)	(±)

+: soluble at room temperature; (+): soluble at heating; (–): insoluble at heating; (±): partially soluble at heating, 0.1% *w*/*v*.

**Table 2:** Transition temperatures (°C) and enthalpy changes (KJ mol^−1^) of the azomethine dimers (DArHn) and compound (MAtH8).

Code	PLM^a^		DSC^b^	
	1H	1C	1H	1C
DAH4	Cr 195 I	I 160 Cr	Cr 192 (40.8)^f^ I	I 149 (34.1) Cr
DAH6	Cr 168 I	I 160 Cr	Cr 166 (45.9) I	I 142 (45.5) Cr
DAH8	Cr 163 I	I 135 Cr	Cr 159 (71.6) I	I 136 (68.9) Cr
DAH9	Cr 102 I	I 79 Cr	Cr 100 (75.2) I	I 77 (73.5) Cr
DNH6	Cr 169 I	–^c^	Cr_1_ 139 Cr_2_ 168 (36.5)^g^ I	I 20 G
DNH8	Cr 128 I	–	Cr_1_ 109 Cr_2_ 125 (61.0)^g^ I	I 9 G
DPyH8	Cr 194 I	I 165 N 137 Cr	Cr 190 (17.6) I	I 160 (0.8) N 127 (11.7) Cr
DPyH9	Cr 136 I	I 108 N –^e^	Cr 134 (38.1) I	I 98 (0.3) N 38 G
DPyH10	Cr 157 I	I 147 N –	Cr_1_120 Cr_2_ 135 Cr_3_ 152 (20.7)^g^ I	I 139 (1.5) N 68 G
MAtH8	Cr_1_ 152 Cr_2_ 170 N 254 I	I 245 N 170 Cr_2_ 120 Cr_1_	Cr_1_ 157 (18.2) Cr_2_ 174 (0.3) N 250 (24.5) I	I 224 (26.0) N 164 (0.5) Cr_2_ 119 (13.2) Cr_1_
DAtH8	Cr 267 I	I 271 N 238 Cr	Cr_1_ 259 Cr_2_ 264 (118.7)^g^ I	I 247 (2.1) N 215 (90.58)Cr
DAtH9	Cr 248 N 260 I	I 255 N 238 Cr	Cr 240 (54.9) N 252 (0.9) I	I 247 (1.2) N 188 (43.9) Cr

1H: first heating scan, 1C: first cooling scan; Cr—crystalline, N—nematic, I—isotropic, G—glass transition; ^a^ transition temperatures noted by polarized light optical microscopy; ^b^ transition temperatures recorded by differential scanning calorimetry; ^c^ undetected transition by PLM; ^e^ frozen phase; ^f^ enthalpy value (in brackets); ^g^ cumulative enthalpy value due to overlapping of transition peaks.

**Table 3:** Photophysical characteristics of the azomethine dimers (DArHn) and compound (MAtH8), in *N*-dimethylformamide (DMF) solution.

Code	λ _abs_ (nm)	λ _edge_ (nm)	ε (M^−1^cm^−1^)	*E_g_* (eV)	λ _em_ (nm)
DAH4	293; **320**	388	2090	3.19	361
DAH6	293; **320**	386	2740	3.21	361
DAH8	294; **320**	386	1610	3.21	361
DAH9	293; **320**	386	1550	3.21	361
DNH6	280; 302; **344**	428	1390	2.89	418
DNH8	280; 303; **344**	428	1690	2.89	415
DPyH8	284; 297; 310; 340; 370; **385**; 410	470	2600	2.63	439
DPyH9	284; 297; 307; 340; 360; **380**; 410	470	4820	2.63	436
DPyH10	285; 297; 310; 340; 370; **385**; 410	470	2530	2.63	439
DAtH8	325; **336**; 364; 390; 411; 437	465	980	2.66	442; 470; 501
DAtH9	294; 307; 321; **335**; 376; 390	465	2280	2.66	379; 406; 445; 490
MAtH8	285; 293; 307; 320; **335**; 375; 390	465	1750	2.66	380; 406; 439; 483

λ_abs_: wavelength of the maximum absorption bands; λ_edge_: wavelength of the absorption edge (onset) of the optical absorption spectra; ε: wavelength-dependent molar absorptivity coefficient; E_g_: energy band gap; λ_em_: Emission wavelength after irradiation with chromophore-imine frame absorption wavelength (bold λ_abs_).

## Data Availability

Data set presented in this study is available in this article or on request from the corresponding authors.

## Sample Availability

Samples of the compounds are available on request from the corresponding authors.
